# Supplementary figures and images for: Genomic analysis for the prediction of prognosis in small-bowel cancer
Source: PLoS One. 2021 May 20;16(5):e0241454. doi: 10.1371/journal.pone.0241454 (PMC8136681; doi:10.1371/journal.pone.0241454)

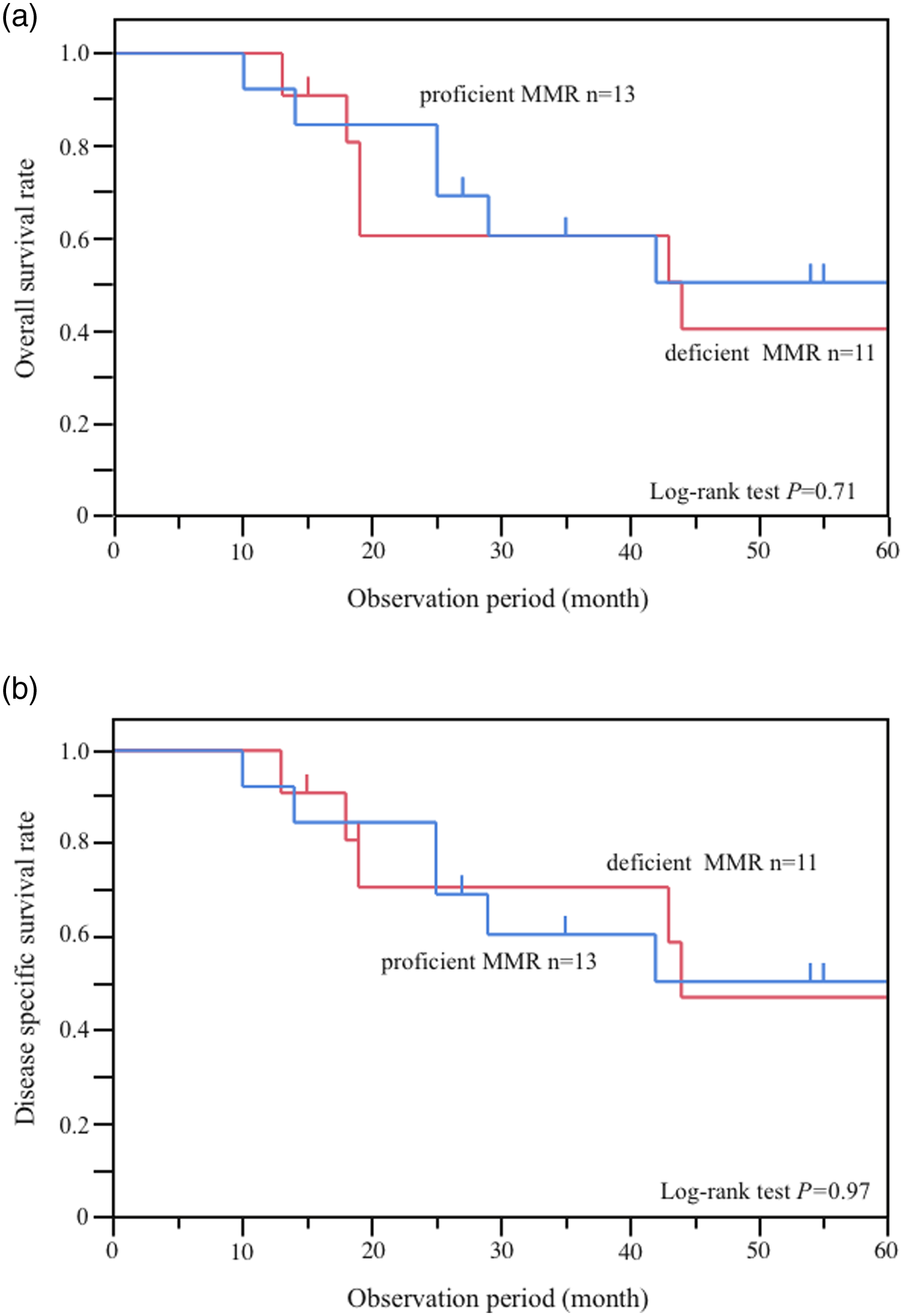

Supplement: S1 Fig — (a) OS of patients with small-bowel cancer related to MMR status. (b) DSS of patients with small-bowel cancer related to the MMR status. There were no significant differences in survival associated with MMR status. (TIFF) [file pone.0241454.s001.tiff]

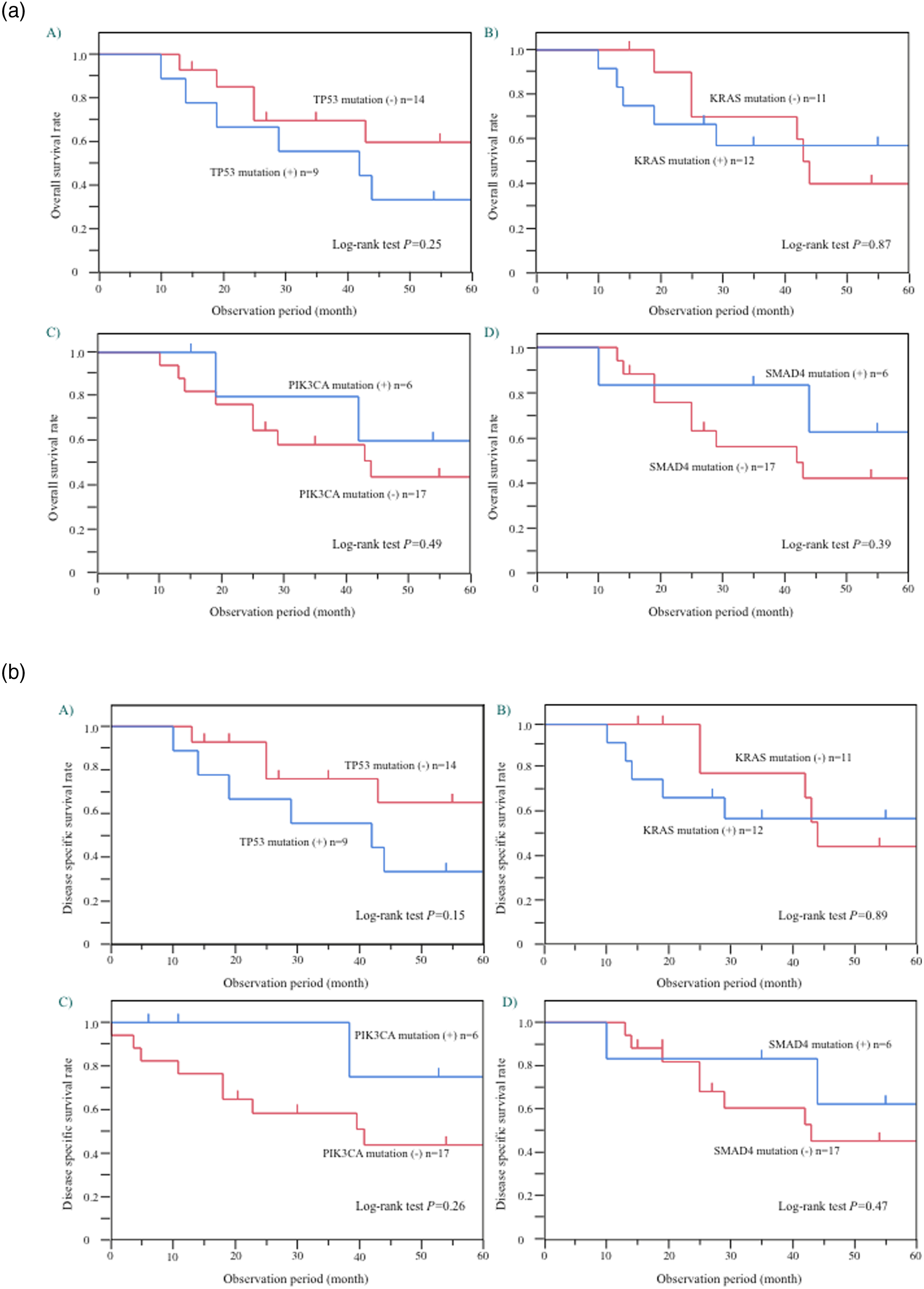

Supplement: S2 Fig — (a) OS of patients with small-bowel cancer and mutations in TP53 (A), KRAS (B), PIK3CA (C), and SMAD4 (D). (b) DSS of patients with small-bowel cancer and mutations in TP53 (A), KRAS (B), PIK3CA (C), and SMAD4 (D). There were no significant differences in survival associated with any gene mutation. (TIFF) [file pone.0241454.s002.tiff]

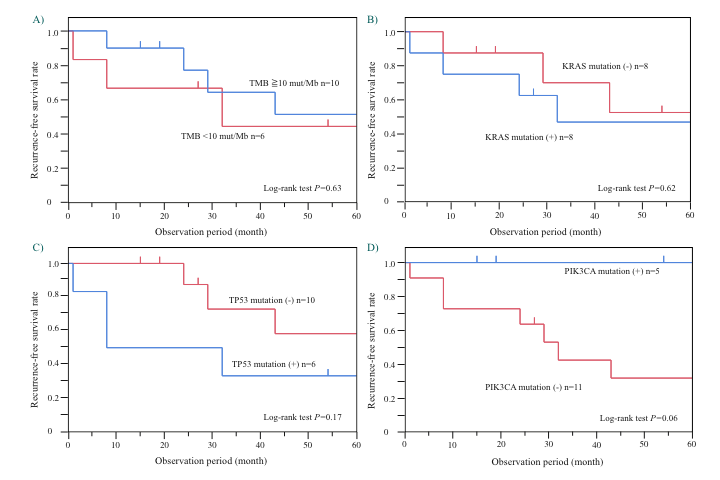

Supplement: S3 Fig — RFS of patients with small-bowel cancer related to tumor mutational burden (TMB; A) and mutations in KRAS (B), TP53 (C), and PIK3CA (D). There were no significant differences in RFS associated with TMB or any gene mutation. (TIFF) [file pone.0241454.s003.tiff]

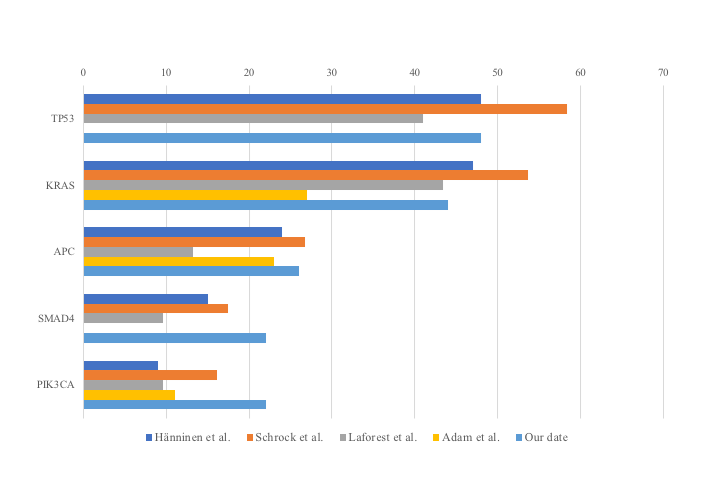

Supplement: S4 Fig — We compared the frequency of genomic variants in TP53, KRAS, APC, SMAD4, and PIK3CA for small-bowel cancers. There were no significantly different genomic variants for any gene. However, the frequency of genomic variant of APC and PIK3CA in this study tended to be higher than that reported in previous studies. (TIFF) [file pone.0241454.s004.tiff]
