# Supplementary material for: Genomic analysis for the prediction of prognosis in small-bowel cancer
Source: PLoS One. 2021 May 20;16(5):e0241454. doi: 10.1371/journal.pone.0241454 (PMC8136681; doi:10.1371/journal.pone.0241454)
Supplement: S1 Table — (DOCX) [file pone.0241454.s005.docx]

**S1 Table. Target panel of 90 cancer-related genes used in our study.**

| *ABL1* | *CREBBP* | *IGF1R* | *NOTCH1* | *ROS1* |
| --- | --- | --- | --- | --- |
| *AKT1* | *CTNNB1* | *IGF2* | *NOTCH2* | *SETD2* |
| *AKT2* | *CUL3* | *IL7R* | *NOTCH3* | *SMAD4* |
| *AKT3* | *DDR2* | *JAK1* | *NRAS* | *SMADRCA4* |
| *ALK* | *EGFR* | *JAK2* | *NRG1* | *SMO* |
| *APC* | *ENO1* | *JAK3* | *NT5C2* | *STAT3* |
| *ARIDIA* | *EP300* | *KEAP1* | *PALB2* | *STK11* |
| *ARID2* | *ERBB2* | *KIT* | *PBRM1* | *TP53* |
| *ATM* | *ERBB3* | *KRAS* | *PDGFRA* | *TSC1* |
| *AXIN1* | *ERBB4* | *MAP2K1* | *PDGFRB* | *VHL* |
| *BAP1* | *EZH2* | *MAP2K4* | *PIK3CA* |  |
| *BARD1* | *FBXW7* | *MAP3K1* | *PIK3R1* |  |
| *BCL2L11* | *FGFR1* | *MAP3K4* | *PTCH1* |  |
| *BRAF* | *FGFR2* | *MDM2* | *PTEN* |  |
| *BRCA1* | *FGFR3* | *MET* | *RAC1* |  |
| *BRCA2* | *FGFR4* | *MTOR* | *RAC2* |  |
| *CCDND1* | *FLT3* | *MYC* | *RAD51C* |  |
| *CD4* | *HRAS* | *MYCN* | *RAF1* |  |
| *CDKN2A* | *IDH1* | *NF1* | *RB1* |  |
| *CHEK2* | *IDH2* | *NFE2L2* | *RET* |  |
