# Supplementary material for: Genomic analysis for the prediction of prognosis in small-bowel cancer
Source: PLoS One. 2021 May 20;16(5):e0241454. doi: 10.1371/journal.pone.0241454 (PMC8136681; doi:10.1371/journal.pone.0241454)
Supplement: S2 Table — (DOCX) [file pone.0241454.s006.docx]

**S2 Table. Outcome of immunohistochemistry analysis.**

| IHC result | Small-bowel cancer (n = 29) |
| --- | --- |
| MMR status |  |
| Absent MLH1 and PMS2 | 7 (24) |
| Absent PMS2 | 0 (0) |
| Absent MSH2 and MSH6 | 3 (10) |
| Absent MSH6 | 3 (10) |
| dMMR | 13 (45) |

Data represented as n (%).

IHC: immunohistochemistry, MMR: mismatch repair protein, dMMR: deficient mismatch repair protein.
