# Supplementary material for: Genomic analysis for the prediction of prognosis in small-bowel cancer
Source: PLoS One. 2021 May 20;16(5):e0241454. doi: 10.1371/journal.pone.0241454 (PMC8136681; doi:10.1371/journal.pone.0241454)
Supplement: S3 Table — (DOCX) [file pone.0241454.s007.docx]

**S3 Table. Characteristics of lesions with high TMB and low TMB.**

| Variable | TMB < 10 mutations/Mb (n = 9) | TMB ≥ 10 mutations/Mb (n = 18) | *P*-Value |
| --- | --- | --- | --- |
| Location |  |  |  |
| Jejunum | 7 (78) | 16 (89) | 0.58 |
| Ileum | 2 (22) | 2 (11) |  |
| Endoscopic stricture | 6 (75) | 6 (33) | 0.13 |
| Tumor diameter, mm, mean ± SD | 41.9 ± 15.1 | 44.1 ± 20.7 | 0.78 |
| Histology |  |  |  |
| tub/pap | 8 (89) | 14 (78) | 0.64 |
| por/sig/muc | 1 (11) | 4 (22) |  |
| Tumor morphology |  |  |  |
| Protruded type | 3 (33) | 0 (0) | 0.03 |
| Depressed type | 6 (67) | 18 (100) |  |
| Pathological stage |  |  |  |
| T0–2/T3–4 | 0/9 | 1/17 | 1.00 |
| N0/N1–3 | 4/5 | 8/10 | 1.00 |
| M0/M1 | 6/3 | 12/6 | 1.00 |
| Pathological staging |  |  |  |
| Stage I–II | 2 (22) | 6 (33) | 0.68 |
| Stage III–IV | 7 (78) | 12 (67) |  |

Data represented as n (%) and mean ± SD.

SD: standard deviation, TMB: tumor mutational burden, tub: tubular adenocarcinoma, pap: papillary adenocarcinoma, por: poorly differentiated adenocarcinoma, sig: signet-ring cell carcinoma, muc: mucinous adenocarcinoma.
