# Supplementary material for: Genomic analysis for the prediction of prognosis in small-bowel cancer
Source: PLoS One. 2021 May 20;16(5):e0241454. doi: 10.1371/journal.pone.0241454 (PMC8136681; doi:10.1371/journal.pone.0241454)
Supplement: Attachment — Submitted filename: Response to Reviewers.docx [file pone.0241454.s008.docx]

September 29, 2020

Dear Editor-in-chief:

We would like to thank you and the reviewers for the thorough review and insightful comments on our manuscript “**Genomic analysis for the prediction of prognosis in small-bowel cancer**” (manuscript ID: PONE-D-20-24291). We have carefully considered all the reviewers’ comments and suggestions, and we have made the corresponding revisions to the manuscript. Additionally, we have carefully proofread the revised manuscript to ensure that the text is optimally phrased and free from typographical and grammatical errors.

We feel that our revisions are suitable in light of the reviewers’ comments, and we believe the revised paper is significantly improved compared to the paper we initially submitted. We hope that this revised version is now acceptable for publication as an Original Article in PLOS ONE*.*

Please do not hesitate to let us know whether there are any further changes we should make to the manuscript.

Yours sincerely,

Akiyoshi Tsuboi, MD

E-mail: atsuboi@hiroshima-u.ac.jp

All related correspondence should be sent to: Shiro Oka, MD, PhD.

Department of Gastroenterology and Metabolism, Graduate School of Biomedical Sciences, Hiroshima University, 1-2-3 Kasumi, Minami-ku, Hiroshima 734-8551, Japan

Telephone: 81-82-257-5193, Fax: 81-82-257-5193

E-mail: oka4683@hiroshima-u.ac.jp

Editorial office:

1) Thank you for stating the following in the Acknowledgments Section of your manuscript:

"This work was supported by the Program of the network-type Joint Usage/Research Center for Radiation Disaster Medical Science of Hiroshima University, Nagasaki University, and Fukushima Medical University. This work does not have any financial interests to disclose."

We note that you have provided funding information that is not currently declared in your Funding Statement. However, funding information should not appear in the Acknowledgments section or other areas of your manuscript. We will only publish funding information present in the Funding Statement section of the online submission form.

Please remove any funding-related text from the manuscript and let us know how you would like to update your Funding Statement. Currently, your Funding Statement reads as follows:

"The authors received no specific funding for this work."

**Reply:** Thank you for your suggestion. Although, we have borrowed inspection equipment, this work did not have any financial interests as noted in the Acknowledgements section. Hence, we have not changed the acknowledgments section and Funding Statement.

2) Thank you for your clarification on the opt-out consent for the use of tissue samples and medical records used in your study. Please explain how patients were informed of this option, and whether the ethics committees listed specifically approved opt-out consent for the use of medical records and tissue samples. Thank you.

**Reply:** Thank you for your suggestion. We changed in Methods section as follows

(p.6 lines 105–113)

From “The study was performed in accordance with the ethical standards of the Declaration of Helsinki and was approved by the Independent Ethics Committee of Hiroshima University (approval number: E-1407; registration date: October 26, 2018) and National Hospital Organization Kure Medical Center and Chugoku Cancer Center (approval number: 2019-10; registration date: May 31, 2019). Regarding informed consent, we provided patients the opportunity to opt-out of the study by posting and presenting details of the study on our website. None of the patients refused inclusion during the study period.”

to “The study was performed in accordance with the ethical standards of the Declaration of Helsinki. The study design and protocol were approved by the Independent Ethics Committee of Hiroshima University (approval number: E-1407; registration date: October 26, 2018) and National Hospital Organization Kure Medical Center and Chugoku Cancer Center (approval number: 2019-10; registration date: May 31, 2019). Written informed consent was obtained from all patients for the use of medical records and tissue samples. Regarding informed consent, we provided patients the opportunity to opt-out of the study by posting and presenting details of the study on our website. None of the patients refused inclusion during the study period.”

Editorial office:

1) Thank you for providing further information about the funding for this study. Please note that even information about provision of materials should be included only in the Financial Disclosure statement and not the Acknowledgments section of your paper. As this is the case, please:

a) Remove the funding information from your manuscript.

b) Confirm that we may update your Financial Disclosure statement to the following:

"This work was supported by the Program of the network-type Joint Usage/Research Center for Radiation Disaster Medical Science of Hiroshima University, Nagasaki University, and Fukushima Medical University. The funder had no role in study design, data collection and analysis, decision to publish, or preparation of the manuscript."

**Reply:** Thank you for your suggestion. We removed acknowledgments section and confirmed changing in the Financial Disclosure statement.

Reviewer #1: The authors present a genomic analysis of small bowel cancer in a retrospective study. The findings indicating that TMB-high patients had improved survival is notable; however, many other conclusions are limited by the small sample size of 24 patients, all of whom underwent surgical resection and did not include duodenal cancers.

-If data on patient treatment (e.g. adjuvant therapy) were included, it would strengthen the novelty of the manuscript.

**Reply:** Thank you for pointing out this matter. We have included adjuvant therapy in Table 1a under ‘Diagnostic modality’ as follows:

**“Table 1** Baseline characteristics of small-bowel cancer

1a. Characteristics patients with small-bowel cancer in our study

| Variables | Small-bowel cancer (n = 24) |
| --- | --- |
| Age (years), mean ± SD | 61.7 ± 11.7 |
| Sex, male/female | 16/8 |
| BMI, mean ± SD | 22.3 ± 6.3 |
| Observation period (month), mean ± SD | 50.0 ± 43.3 |
| Chief complaint, +/− | 22/2 |
| Intestinal obstruction | 9 (38) |
| Abdominal pain | 8 (33) |
| Gastrointestinal bleeding | 5 (21) |
| History of other organ cancers | 7 (29) |
| Colorectal cancer | 3(13) |
| Breast cancer | 2(8) |
| Gastric cancer | 1(4) |
| Lung cancer | 1(4) |
| Bladder cancer | 1(4) |
| Pancreatic cancer | 1(4) |
| Endometrial cancer | 1(4) |
| Gallbladder cancer | 1(4) |
| Predisposing conditions |  |
| FAP | 0 (0) |
| PJS | 0 (0) |
| LS | 2 (8) |
| Crohn’s disease | 0 (0) |
| Celiac disease | 0 (0) |
| Number of lesions, single/ multiple | 21/3 |
| CEA, median [IQR] | 2.2 ng/mL [1.4–5.9] |
| CA19-9, median [IQR] | 8.5 U/mL [2–77.9] |
| Diagnostic modality |  |
| DBE | 20 (83) |
| CT | 3 (13) |
| CE | 1 (4) |
| Adjuvant therapy |  |
| TS1 | 5 (21) |
| FOLFOX | 4 (17) |
| CapeOX | 3 (13) |
| Capecitabine | 2 (8) |
| UFT/LV | 2 (8) |
| FOLFOX+Bmab | 1 (4) |
| TS1+Doc | 1 (4) |
| None | 6 (25) |

Data represented as n (%) and mean ± SD.

SD: standard deviation, BMI: body mass index, FAP: familial adenomatous polyposis, PJS: Peutz-Jeghers syndrome, LS: Lynch syndrome, CEA: carcinoembryonic antigen, CA19-19: carbohydrate antigen 19-9, IQR: interquartile range, DBE: double balloon endoscopy, CT: computed tomography, CE: capsule endoscopy”

A total of 18 of the 24 patients received postoperative adjuvant chemotherapy and the regimens were as follows: 5 patients received TS-1, 4 patients received FOLFOX, 3 patients received CapeOX, 2 patients received Capecitabine, 2 patients received UFT/LV, 1 patient received FOLFOX+B-mab, and 1 patient received TS-1+ Docetaxel. Postoperative adjuvant chemotherapy was not correlated with OS (HR, 2.47; 95% CI, 0.64–16.16; *P* = 0.20) (Table 3). All patients (6/6) with TMB <10 mut/Mb received postoperative adjuvant chemotherapy, while 65% of the patients (11/17) with TMB ≥10 mut/Mb received postoperative adjuvant chemotherapy. All patients (6/6) with TMB <10 mut/Mb that received postoperative adjuvant chemotherapy had a worse prognosis than those with TMB ≥10 mut/Mb. Therefore, we believe that cancer genomic analysis of resected specimens may allow for indication of postoperative adjuvant chemotherapy depending on the value of TMB.

-The genetics were similar to Western data, why do the offers suggest that small bowel cancer has a different biology in the Japanese population?

**Reply:** We probably misled you when expressing our opinion without giving it much thought. The frequencies of redisposing factors of small-bowel cancer such as FAP, LS, Crohn’s disease, celiac disease, and BMI are different in Japanese and Caucasian populations. Thus, we suggest that these factors may have an effect on the development of small-bowel cancer. The genetics were indeed similar to that of Western data; however, some difference was found in the frequencies of APC mutations between our data and previous reports from Western countries.

We have altered some sentences in the revised version of the paper as follows:

(p.5 lines 81–88)

From “However, significantly different factors are involved the development of small-bowel cancer exist between Caucasian and Japanese populations.”

To “However, disease structure is different between Caucasian and Japanese populations. For example, background factors such as the frequency of celiac disease, Crohn's disease, and obesity are different between Japan and Western countries. The frequency of obesity varies across countries. According to World Health Organization (2016), a 20–40% frequency of body mass index (BMI) >30 was reported in Western countries, whereas a 4.4% frequency of BMI >30 was reported in Japan; this scenario is explained by Japanese people tending to consume diets low in fat, sugar and fructose.”

-A more rigorous statistical analysis is necessary to elucidate the contributions of TMB and SMAD4 over known prognostic factors such as R0 resection rate, stage, etc. using a Cox proportional hazards model.

**Reply:** Thank you for pointing this out. After re-examining the Cox proportional hazards model, we found that Stage and TMB were correlated with OS, and that Stage and SMAD4 were correlated with RFS. These results suggest that the presence of SMAD4 mutations and TMB status are strongly correlated. These results are presented in Tables 3–6) as follows:

(p. 17–19)

**Table 3** Univariate association with overall survival

| Variables | | N | OS | | |
| --- | --- | --- | --- | --- | --- |
|  |  |  | HR (95%CI) | *P*-value | Log-rank  *P*-value |
| Sex | Male | 16 | 2.60 (0.68–16.94) | 0.17 | 0.20 |
|  | Female | 8 | Ref. |  |  |
| Age | >65 | 8 | 0.86 (0.23–2.73) | 0.80 | 0.80 |
|  | ≦65 | 16 | Ref. |  |  |
| pStage | IV | 9 | 12.13 (2.89–83.36) | <0.01 | <0.01 |
|  | I–III | 15 | Ref. |  |  |
| R0 resection | R1/2 | 8 | 7.7 (2.06–37.25) | <0.01 | <0.01 |
|  | R0 | 16 | Ref. |  |  |
| Adjuvant chemotherapy | Done | 18 | 2.47 (0.64–16.16) | 0.20 | 0.23 |
|  | None | 6 | Ref. |  |  |
| IHC | dMMR | 11 | 1.25 (0.38–3.99) | 0.71 | 0.71 |
|  | pMMR | 13 | Ref. |  |  |
| TMB | <10 | 6 | 3.29 (0.84–11.24) | 0.08 | <0.05 |
|  | ≧10 | 17 | Ref. |  |  |
| SMAD4 | Mutation | 6 | 0.52 (0.08–2.01) | 0.37 | 0.39 |
|  | Wild | 17 | Ref. |  |  |

Data represented as n.

IHC: immunohistochemical, TMB: tumor mutational burden, mut/MB: mutations per megabyte of DNA, MMR: mismatch repair, dMMR: deficient mismatch repair, pMMR: proficient mismatch repair, HR: hazard ration, OS: overall survival

(p. 18)

**Table 4** Multivariate association with overall survival

| Variables | | N | OS | |
| --- | --- | --- | --- | --- |
|  |  |  | HR (95%CI) | *P*-value |
| pStage | IV | 9 | 58.68 (7.89–1348.14) | <0.01 |
|  | I–III | 15 | Ref. |  |
| TMB | <10 | 6 | 11.33 (2.08–85.05) | <0.01 |
|  | ≧10 | 17 | Ref. |  |

Data represented as n.

TMB: tumor mutational burden, mut/MB: mutations per megabyte of DNA, HR: hazard ration, OS: overall survival

(p. 20)

**Table 5** Univariate association with recurrence-free survival

| Variables | | N | RFS | | |
| --- | --- | --- | --- | --- | --- |
|  |  |  | HR (95%CI) | *P*-value | Log-rank  *P*-value |
| Sex | Male | 10 | 2.98 (0.50–56.65) | 0.26 | 0.29 |
|  | Female | 6 | Ref. |  |  |
| Age | >65 | 7 | 1.21 (0.23–5.61) | 0.81 | 0.81 |
|  | ≦65 | 9 | Ref. |  |  |
| pStage | IV | 3 | 11.43 (1.09–247.04) | 0.04 | 0.01 |
|  | I–III | 13 | Ref. |  |  |
| Adjuvant chemotherapy | Done | 10 | 1.74 (0.38–12.22) | 0.49 | 0.50 |
|  | None | 6 | Ref. |  |  |
| IHC | dMMR | 8 | 0.69 (0.14–3.13) | 0.62 | 0.62 |
|  | pMMR | 8 | Ref. |  |  |
| TMB | <10 | 3 | 2.97 (0.41–15.37) | 0.25 | 0.18 |
|  | ≧10 | 13 | Ref. |  |  |
| SMAD4 | Mutation | 5 | 4.15 (0.90–21.41) | 0.07 | 0.04 |
|  | Wild | 11 | Ref. |  |  |

Data represented as n.

IHC: immunohistochemical, TMB: tumor mutational burden, mut/MB: mutations per megabyte of DNA, MMR: mismatch repair, dMMR: deficient mismatch repair, pMMR: proficient mismatch repair, HR: hazard ration, OS: overall survival

(p. 20–21)

**Table 6** Multivariate association with recurrence-free survival

| Variables | | N | OS | |
| --- | --- | --- | --- | --- |
|  |  |  | HR (95%CI) | *P*-value |
| pStage | IV | 3 | 24.07 (1.84–654.62) | 0.02 |
|  | I–III | 13 | Ref. |  |
| TMB | <10 | 5 | 6.72 (1.28–49.54) | 0.03 |
|  | ≧10 | 11 | Ref. |  |

Data represented as n.

TMB: tumor mutational burden, mut/MB: mutations per megabyte of DNA, HR: hazard ration, RFS: recurrence-free survival

Moreover, we included the following text in the Results section:

(p. 17 lines 290–297)

“The other covariates from the Cox proportional hazard analysis associated with OS are shown in Tables 3 and 4. Regarding the univariate analysis, pathological Stage IV (HR, 12.13; 95% CI, 2.89–83.36; *P* < 0.01) and R1/2 resection (HR, 7.7; 95% CI, 2.06–37.25; *P* < 0.01) were correlated with OS (Table 3), whereas TMB ≥10 mutations/Mb was not significantly correlated with OS (HR, 3.29; 95% CI, 0.84–11.24; *P* = 0.08). However, in the multivariate analysis, pathological Stage IV (HR, 58.68; 95% CI, 7.89–1348.14; *P* < 0.01) and TMB ≥10 mutations/Mb (HR, 11.33; 95% CI, 2.08–85.05; *P* < 0.01) were correlated with OS.”

(p. 19 lines 315–321)

“The other covariates from the Cox proportional hazard analysis associated with RFS are shown in Tables 5 and 6. Regarding the univariate analysis, pathological Stage IV (HR, 11.43; 95% CI, 1.09–247.04; *P* = 0.04) was correlated with RFS (Table 5), whereas mutated *SMAD4* was not significantly correlated with RFS (HR, 4.15; 95% CI, 0.90–21.41; *P* = 0.07). However, pathological Stage IV (HR, 24.07; 95% CI, 1.84–654.62; *P* = 0.02) and mutated *SMAD4* (HR, 6.72; 95% CI, 1.28–49.54; *P* = 0.03) were correlated with RFS in the multivariate analysis.”

-Breast is misspelled in Table 1.

**Reply:** Thank you for pointing this out. I corrected the spelling in Table 1.

Reviewer #2: This is a well-written manuscript on a very interesting topic in a highly selected population of small-bowel cancer (duodenum not included).

The main issue here is the number of patients included in the analysis, which make results quite "weak" and not robust enough to be reliable.

Major:

-TMB cut off: please clarify why 10 is used as cutoff.

**Reply:** Thank you for pointing that out. The problem here is that the cut-off value of TMB for small-bowel cancer has not yet been determined. However, Shrock et al. (JAMA Oncol. 2017; 3: 1546-1553.) defined the TMB status of <10 as low, 10–20 as intermediate, and 20> as high. Therefore, in the present study, we defined TMB ≥10 mutations/Mb as being a high value.

-MMR deficiency rates are higher then expected, sure this may have an impact both in TMB and in OS data. Please clarify this in the manuscript.

**Reply:** Thank you for pointing this out. The Cox proportional hazards model showed that dMMR was not correlated with OS, but the stepwise method showed that Stage and TMB were correlated with OS. We describe this in the Results section:

(p. 17 lines 290–297)

“The other covariates from the Cox proportional hazard analysis associated with OS are shown in Tables 3 and 4. Regarding the univariate analysis, pathological Stage IV (HR, 12.13; 95% CI, 2.89–83.36; *P* < 0.01) and R1/2 resection (HR, 7.7; 95% CI, 2.06–37.25; *P* < 0.01) were correlated with OS (Table 3), whereas TMB ≥10 mutations/Mb was not significantly correlated with OS (HR, 3.29; 95% CI, 0.84–11.24; *P* = 0.08). However, in the multivariate analysis, pathological Stage IV (HR, 58.68; 95% CI, 7.89–1348.14; *P* < 0.01) and TMB ≥10 mutations/Mb (HR, 11.33; 95% CI, 2.08–85.05; *P* < 0.01) were correlated with OS.”

(p. 17–19)

**Table 3** Univariate association with overall survival

| Variables | | N | OS | | |
| --- | --- | --- | --- | --- | --- |
|  |  |  | HR (95%CI) | *P*-value | Log-rank  *P*-value |
| Sex | Male | 16 | 2.60 (0.68–16.94) | 0.17 | 0.20 |
|  | Female | 8 | Ref. |  |  |
| Age | >65 | 8 | 0.86 (0.23–2.73) | 0.80 | 0.80 |
|  | ≦65 | 16 | Ref. |  |  |
| pStage | IV | 9 | 12.13 (2.89–83.36) | <0.01 | <0.01 |
|  | I–III | 15 | Ref. |  |  |
| R0 resection | R1/2 | 8 | 7.7 (2.06–37.25) | <0.01 | <0.01 |
|  | R0 | 16 | Ref. |  |  |
| Adjuvant chemotherapy | Done | 18 | 2.47 (0.64–16.16) | 0.20 | 0.23 |
|  | None | 6 | Ref. |  |  |
| IHC | dMMR | 11 | 1.25 (0.38–3.99) | 0.71 | 0.71 |
|  | pMMR | 13 | Ref. |  |  |
| TMB | <10 | 6 | 3.29 (0.84–11.24) | 0.08 | <0.05 |
|  | ≧10 | 17 | Ref. |  |  |
| SMAD4 | Mutation | 6 | 0.52 (0.08–2.01) | 0.37 | 0.39 |
|  | Wild | 17 | Ref. |  |  |

Data represented as n.

IHC: immunohistochemical, TMB: tumor mutational burden, mut/MB: mutations per megabyte of DNA, MMR: mismatch repair, dMMR: deficient mismatch repair, pMMR: proficient mismatch repair, HR: hazard ration, OS: overall survival

(p. 18)

**Table 4** Multivariate association with overall survival

| Variables | | N | OS | |
| --- | --- | --- | --- | --- |
|  |  |  | HR (95%CI) | *P*-value |
| pStage | IV | 9 | 58.68 (7.89–1348.14) | <0.01 |
|  | I–III | 15 | Ref. |  |
| TMB | <10 | 6 | 11.33 (2.08–85.05) | <0.01 |
|  | ≧10 | 17 | Ref. |  |

Data represented as n.

TMB: tumor mutational burden, mut/MB: mutations per megabyte of DNA, HR: hazard ration, OS: overall survival

-Au say "Although, we did not observe any correlation between DSS and OS, which may be due to the small number of cases analyzed, we believe that the SMAD4 mutations may be a prognostic factor for small-bowel cancer, similar to that of CRC. " Please provide more data and explanations for this statement.

**Reply:** Thank you for pointing this out. The case of recurrence with SMAD4 mutation is relatively recent, and the patient is still undergoing multidisciplinary treatment. However, there are no current cases of CR, which may affect OS and DSS in the future as we perform the long-term follow up. More detailed data are provided in the Results section:

(p. 19 lines 324–329)

“Of the four patients that had recurrence of small-bowel cancer, two patients died from primary cancer, whereas the other two patients are still alive and undergoing treatment with multidisciplinary treatment including additional surgery. Of the two patients that had recurrence at Stage II, one of them received postoperative adjuvant chemotherapy with capecitabine, and the other did not receive adjuvant chemotherapy.”

and in the Discussion section:

(p. 25 lines 412–419)

“Two of the three patients diagnosed at Stage II with SMAD4 mutation had recurrence by peritoneal dissemination. Currently, adjuvant chemotherapy is not recommended for all patients with Stage II CRC in Japan; however, these results suggest that postoperative adjuvant chemotherapy should be considered for Stage II small-bowel cancer with SMAD4 mutations because of the high risk of recurrence. Although no correlation was observed between DSS and OS, possibly because of the small number of cases analyzed, further studies may indicate SMAD4 mutations as a prognostic factor for small-bowel cancer, as in CRC.”
